# Radiological hints for differentiation of cerebellar multiple system atrophy from spinocerebellar ataxia

**DOI:** 10.1038/s41598-022-14531-0

**Published:** 2022-06-22

**Authors:** Hung-Chieh Chen, Li-Hua Lee, Jiing-Feng Lirng, Bing-wen Soong

**Affiliations:** 1 School of Medicine, National Yang Ming Chiao Tung University, Taipei, Taiwan; 2 Department of Radiology, Taichung Veterans General Hospital, Taichung, Taiwan; 3 Department of Neurology, Cardinal Tien Hospital, New Taipei City, Taiwan; 4 Department of Radiology, Taipei Veterans General Hospital, Taipei, Taiwan; 5 Taipei Neuroscience Institute, Taipei Medical University, Taipei, Taiwan; 6 Department of Neurology, Taipei Medical University-Shuang Ho Hospital, New Taipei City, Taiwan; 7 Department of Neurology, Taipei Veterans General Hospital, and Brain Research Center, National Yang Ming Chiao Tung University, Taipei, Taiwan

**Keywords:** Neuroscience, Neurology

## Abstract

Differentiation cerebellar multiple systemic atrophy (MSA-C) from spinocerebellar ataxia (SCA) is important. The “hot cross bun” sign (HCBS) at pons and magnetic resonance spectroscopy (MRS) are helpful. However, the prevalence of HCBS and the alteration of cerebellar MRS parameters are evolving with disease progression. We hypothesized that since the HCBS and MRS are evolving with time, different parameters for differentiation of MSA-C and SCA are required at different disease stages. The aim of this study was to evaluate the HCBS and MRS changes in patients with MSA-C and SCA at different disease stages. A total of 398 patients with molecularly confirmed SCA (SCA1, 2, 3, 6, 17) and 286 patients diagnosed with probable MSA-C (without mutations in SCA1, 2, 3, 6, 17 genes), who had received brain magnetic resonance imaging (MRI) and MRS from January 2000 to January 2020, were recruited. Twenty-five patients were molecularly identified as having SCA1, 68 as SCA2, 253 as SCA3, 34 as SCA6, and 18 as SCA17. We compared their clinical parameters and neuroimaging features at different disease stages. The presence of HCBS was assessed using an axial T2 fast spin-echo or FLAIR sequence. Proton MRS was recorded with voxel of interest focusing on cerebellar hemispheres and cerebellar vermis and avoiding cerebrospinal fluid spaces space using a single-voxel stimulated echo acquisition mode sequence. We found that patients with MSA-C tend to have a higher prevalence of pontine HCBS, worse Scale for the Assessment and Rating of Ataxia scores, lower cerebellar *N*-acetyl aspartate (NAA)/creatinine (Cr), and choline (Cho)/Cr, compared to patients with SCA at corresponding disease stages. In MSA-C patients with a disease duration < 1 year and without pontine HCBS, a cerebellar NAA/Cr ≤ 0.79 is a good indicator of the possibility of MSA-C. By using the pontine HCBS and cerebellar MRS, discerning MSA-C from SCA became possible. This study provides cutoff values of MRS to serve as clues in differentiating MSA-C from SCAs.

## Introduction

Ataxia is a commonly encountered clinical presentation in the neurology clinics, resulting from a large number of hereditary and non-hereditary causes. Among these diseases, differentiation of idiopathic multiple systemic atrophy, cerebellar type (MSA-C), and spinocerebellar ataxia (SCA) is unequivocally important for patients without a family history and for patients in the early stages of the diseases, while ataxia might be the only clinical manifestation. Patients with MSA-C may then be bothered by additional autonomic dysfunction or parkinsonism and would progress faster in the ensuing years.

Brain magnetic resonance imaging (MRI) and MR spectroscopy (MRS) are readily accessible and useful tools in daily neurological practice. A high prevalence of hot cross bun sign (HCBS)^1^ at pons and significantly lower cerebellar and vermis *N*-acetyl aspartate (NAA)/creatinine (Cr) and choline (Cho)/Cr have been reported in patients with MSA-C compared to those with SCAs^2^. However, patients with several subtypes of SCA may also have pontine HCBS and relatively lower NAA/Cr and Cho/Cr ratios in the cerebellum^2,3^. The features of pontine HCBS^4^ and MRS^2^ of the cerebellum are also continuously evolving with disease progression, and these have seldom been discussed in the literature.

We hypothesized that because the HCBS and MRS are evolving with time, different parameters for differentiation of MSA-C and SCA are required at different disease stages. The aim of this study was to evaluate the HCBS and MRS changes in patients with MSA-C or SCA at different disease stages.

## Methods

### Participants

This study was approved by the Institutional Review Board (IRB) of Taipei Veterans General Hospital, Taipei, Taiwan. All procedures were performed in accordance with the relevant guidelines and regulations, and informed consent was approved by the IRB (2018-01-017B) and signed by the patients.

We retrospectively reviewed the medical records of patients who were either molecularly confirmed with SCA or diagnosed with probable MSA-C based on the second consensus criteria^3^ of MSA-C (without mutations in SCA1, 2, 3, 6, 7, 8, and 17 genes) at the neurology department from January 2000 through January 2020. Patients who had received at least one brain MRI and MRS focusing on the cerebellar hemispheres and vermis were included. A total of 398 patients with various genotypes of SCA and 286 patients with MSA-C were recruited. Twenty-five patients were molecularly identified as having SCA1, 68 as SCA2, 253 as SCA3, 34 as SCA6, and 18 as SCA17. Clinical parameters, such as the age at disease onset, sex, the triplet (CAG) repeat length in each SCA subtypes, disease duration at the time when MRI was performed, age at MRI examination, and the Scale for the Assessment and Rating of Ataxia (SARA) scores within 6 months prior to or after the MRI in patients who were diagnosed after 2006 were studied. The disease onset time was defined by the onset of ataxia symptoms of diseases. The demographic features are listed in Table 1.

**Table 1:** Demographics of patients with MSA-C and SCA.

	Total (n = 684)	SCA (n = 398, 59.43%)	MSA-C (n = 286, 40.57%)	*p* value
Age of onset^a^	47.13 ± 13.00	41.4 ± 12.66	55.72 ± 7.71	< 0.001
Age at MRI examination^a^	52.96 ± 12.19	48.81 ± 13.17	58.73 ± 7.56	< 0.001
**Sex**^b^				
Female	365	218	147	0.431
Male	319	180	139	

^a^Mann–Whitney test. ^b^Chi-square test.

Numeric data are presented as mean ± standard deviation.

*MSA-C* multiple system atrophy, cerebellar type; *SCA* spinocerebellar ataxia.

### Neuroimaging and spectroscopic acquisition

Brain MRI and MRS were performed using a 1.5-T system (Signa EXCITE, GE Medical Systems, Milwaukee, WI, USA). The MRI protocol consisted of an axial T1-weighted three-dimensional fast spoiled gradient recalled acquisition in steady state images [repetition time (TR) 8.58 ms, echo time (TE) 3.62 ms, inversion time (TI) 400 ms], an axial T2 fast spin-echo sequence [TR 4000 ms, TE 256.5 ms] and axial fluid attenuated inversion recovery (FLAIR) [TR 5000 ms, TE 350 ms, TI 1800 ms]. The presence of HCBS was assessed by two experienced neuroradiologists (JFL and HCC) according to the criteria reported by Horimoto^5^ using a T2 fast spin-echo or FLAIR sequence. The pontine HCBSs of grades 3 to 5 were considered positive in this study.

After MRI, proton MRS was recorded in the cerebellar hemispheres and cerebellar vermis using a single-voxel stimulated echo acquisition mode sequence [3000/15/13.7/96 (TR/TE/mixing time/excitations), spectral width = 2500 Hz, number of points = 2048, voxel size = 2 cm × 2 cm × 2 cm]. The voxel of interest (VOI) in each subject was uniformly placed by the same investigator (JFL). Care was taken to avoid the cerebrospinal fluid spaces within the VOIs. The peak areas for NAA at 2.02 parts per million (ppm), Cr at 3.03 ppm, and Cho at 3.22 ppm were measured using the Functool provided by MR company (GE XVi, Milwaukee, WI). The peak integral values are expressed relative to the Cr peak.

Metabolite intensity ratios were automatically calculated at the end of each voxel acquisition, including NAA/Cr and Cho/Cr. To ensure high quality, MRS results with full width at half maximum (FWHM) of 6 Hz were disqualified from the MRS analyses.

### Statistical analyses

Comparisons of the age at examination, the age at MRI examination, the disease duration, the metabolic parameters on MRS (NAA/Cr, Cho/Cr in the cerebellar hemispheres, and NAA/Cr, Cho/Cr in the vermis) between patients with MSA-C vs. different SCAs were performed using the nonparametric Mann–Whitney *U* test due to non-Gaussian distribution of the MRS parameters. The relationship between the age of onset and CAG repeat lengths in SCA patients, between disease duration and metabolic ratios on the MRS, and between disease duration and SARA scores in individual patients were correlated using Spearman’s rank test. The receiver operating characteristic (ROC) curve was used to identify the optimal cutoff of MRS to differentiate MSA-C and SCA in different disease stages. Differences were considered significant at p < 0.05.

### Ethics approval

The study protocols were approved by local ethics committees at each of the participating sites and participants provided informed written consent.

## Results

### Patients (Table 1)

The age of onset and the age at MRI examination were significantly younger in patients with SCA than in those with MSA-C. There was no significant difference in sex distribution between these two disease entities.

### The CAG repeat lengths and the presence of the HCBS in different subtypes of SCA and MSA-C (Table 2)

**Table 2:** The presence of HCBS in different subtypes of SCA and MSA-C.

	Total	Length of the expanded CAG repeats	HCBS negative (n = 400, 58.48%)		HCBS positive (n = 284, 41.52%)		*p* value
	N	Mean ± SD (N)	N	%	N	%	
SCA 1	25	46.88 ± 4.67 (25)	24.00	96.00	1.00	4.00	< 0.001
SCA 2	68	41.35 ± 3.94 (68)	48.00	70.59	20.00	29.41	
SCA 3	253	70.64 ± 4.26 (253)	246.00	97.23	7.00	2.77	
SCA 6	34	23.35 ± 1.28 (34)	34.00	100.00	0.00	0	
SCA 17	18	44.81 ± 3.02 (18)	17.00	94.44	1.00	5.56	
MSA-C	286	0	31	10.84	255	89.16	

*HCBS* hot cross bun sign; *MSA-C* multiple system atrophy, cerebellar type; *SCA* spinocerebellar ataxia.

Chi-square test.

Numeric data are presented as mean ± standard deviation.

All SCA patients in this study have pathologically expanded alleles in different genes. However, among the patients with MSA, none have an intermediate or expanded SCA alleles (SCA1, 2, 3, 6, 7, or 17).

Among the 286 patients with MSA-C, 249 (87.1%) had a pontine HCBS on T2WI MRI at the first brain MRI examination. Of the 684 study subjects (398 with SCA and 286 with MSA-C), 107 patients had a follow-up brain MRI/MRS and six MSA-C patients demonstrated novel HCBS later with disease evolution. The overall prevalence of pontine HCBS was 89.16% in patients with MSA-C and 7.29% in patients with SCA, (among them, 68.97% was found to be SCA2).

### Correlation between parameters

The CAG repeat lengths were negatively correlated with the age of onset in SCA patients (p < 0.001). Disease duration was significantly and positively correlated with SARA scores (p < 0.001), and negatively correlated with cerebellar NAA/Cr (p = 0.040), cerebellar Cho/Cr (p < 0.001), vermis NAA/Cr (p = 0.005), and vermis Cho/Cr (p = 0.017). The longer the disease duration, the higher the SARA scores, and the lower the MRS ratios. Because disease duration significantly correlated with MRS parameters and SARA scores, we opted to evaluate the differences in these parameters based on different disease durations.

### The differences of parameters between patients of MSA-C and SCA with different disease duration (Table 3)

**Table 3:** The differences of parameters between patients of MSA-C and SCA with different disease durations.

The differences of parameters between patients with MSA-C and SCA with a disease duration within 1 year					
		Total (N = 132)	SCA (N = 47)	MSA-C (N = 85)	p value
HCBS^b^	Positive		1 (2.13%)	70 (82.35%)	0.000
	Negative		46 (97.87%)	15 (17.65%)	
Disease duration^a^		0.91 ± 0.28	0.87 ± 0.34	0.94 ± 0.24	0.264
Cerebellum^a^	NAA/Cr	0.76 ± 0.18	0.94 ± 0.14	0.66 ± 0.12	0.000
	Cho/Cr	0.65 ± 0.15	0.74 ± 0.17	0.60 ± 0.10	0.000
Vermis^a^	NAA/Cr	0.78 ± 0.10	0.86 ± 0.09	0.73 ± 0.08	0.000
	Cho/Cr	0.64 ± 0.09	0.69 ± 0.09	0.61 ± 0.07	0.000
SARA score^a^		Total (N = 111)	SCA (N = 42)	MSA-C (N = 69)	0.000
		9.48 ± 5.32	6.40 ± 4.22	11.19 ± 5.11	

The differences of parameters between patients with MSA-C and SCA with a disease duration in the bracket of 2–3 years					
		Total (N = 191)	SCA (N = 89)	MSA-C (N = 102)	
HCBS^b^	Positive		8 (8.99%)	95 (93.14%)	0.000
	Negative		81 (91.01%)	7 (6.86%)	
Disease duration^a^		2.51 ± 0.50	2.57 ± 0.50	2.46 ± 0.50	0.120
Cerebellum^a^	NAA/Cr	0.76 ± 0.19	0.88 ± 0.19	0.65 ± 0.11	0.000
	Cho/Cr	0.64 ± 0.13	0.70 ± 0.13	0.59 ± 0.10	0.000
Vermis^a^	NAA/Cr	0.75 ± 0.11	0.81 ± 0.11	0.73 ± 0.08	0.000
	Cho/Cr	0.64 ± 0.10	0.69 ± 0.10	0.60 ± 0.08	0.000
SARA score^a^		Total (N = 163)	SCA (N = 76)	MSA-C (N = 87)	0.000
		11.86 ± 6.47	8.49 ± 5.70	14.76 ± 5.66	

The differences of parameters between patients with MSA-C and SCA with a disease duration in the bracket of 4–5 years					
		Total (N = 160)	SCA (N = 61)	MSA-C (N = 99)	
HCBS^b^	Positive		3 (4.92%)	96 (96.97%)	0.000
	Negative		58 (95.08%)	3 (3.03%)	
Disease duration^a^		4.49 ± 0.51	4.62 ± 0.49	4.41 ± 0.50	0.009
Cerebellum^a^	NAA/Cr	0.69 ± 0.1863 +	0.85 ± 0.15	0.59 ± 0.11	0.000
	Cho/Cr	0.60 ± 0.14	0.68 ± 0.11	0.54 ± 0.13	0.000
Vermis^a^	NAA/Cr	0.71 ± 0.13	0.82 ± 0.09	0.64 ± 0.10	0.000
	Cho/Cr	0.61 ± 0.11	0.69 ± 0.08	0.57 ± 0.09	0.000
SARA score^a^		Total (N = 134)	SCA (N = 53)	MSA-C (N = 81)	0.000
		16.43 ± 7.06	11.61 ± 4.07	19.52 ± 6.84	

The differences of parameters between patients with MSA-C and SCA with a disease duration in the bracket of 6–8 years					
		Total (N = 151)	SCA (N = 88)	MSA-C (N = 63)	
HCBS^b^	Positive		11 (12.50%)	61 (96.83%)	0.000
	Negative		77 (87.50%)	2 (3.17%)	
Disease duration^a^		6.77 ± 0.78	6.69 ± 0.76	6.87 ± 0.81	0.159
Cerebellum^a^	NAA/Cr	0.72 ± 0.21	0.83 ± 0.18	0.56 ± 0.13	0.000
	Cho/Cr	0.60 ± 0.15	0.68 ± 0.11	0.48 ± 0.11	0.000
Vermis^a^	NAA/Cr	0.73 ± 0.13	0.81 ± 0.09	0.62 ± 0.09	0.000
	Cho/Cr	0.61 ± 0.13	0.68 ± 0.10	0.52 ± 0.10	0.000
SARA score^a^		Total (N = 132)	SCA (N = 80)	MSA-C (N = 52)	0.000
		16.50 ± 8.44	11.99 ± 5.50	22.99 ± 7.70	

The differences of parameters between patients with MSA-C and SCA with a disease duration longer than 8 years					
		Total (N = 157)	SCA (N = 127)	MSA-C (N = 30)	
HCBS^b^	Positive		13 (10.24%)	29 (96.67%)	0.000
	Negative		114 (89.76%)	1 (3.33%)	
Disease duration^a^		12.50 ± 4.94	13.24 ± 5.17	9.38 ± 1.60	0.000
Cerebellum^a^	NAA/Cr	0.74 ± 0.17	0.78 ± 0.15	0.54 ± 0.12	0.000
	Cho/Cr	0.62 ± 0.13	0.65 ± 0.11	0.47 ± 0.11	0.000
Vermis^a^	NAA/Cr	0.74 ± 0.14	0.76 ± 0.13	0.63 ± 0.10	0.000
	Cho/Cr	0.62 ± 0.11	0.65 ± 0.10	0.52 ± 0.09	0.000
SARA score^a^		Total (N = 128)	SCA (N = 103)	MSA-C (N = 25)	0.000
		17.96 ± 8.95	16.20 ± 8.25	25.20 ± 8.20	

^a^Mann-Whitney test. ^b^Chi-square test.

Numeric data are presented as mean ± standard deviation.

*HCBS* hot cross bun sign; *MSA-C* multiple system atrophy, cerebellar type; *SCA* spinocerebellar ataxia; *SARA* Scale for the Assessment and Rating of Ataxia; *NAA* *N*-acetyl aspartate; *Cr* creatinine; *Cho* Choline.

For patients with a disease duration within 1 year, despite their comparable severity of clinical symptoms, the prevalence of HCBS was significantly higher in patients with MSA-C than in those with SCA. Significantly lower cerebellar NAA/Cr, Cho/Cr, and vermis NAA/Cr, Cho/Cr, and higher SARA scores were also found in patients with MSA-C.

Using receiver operating characteristic curve (ROC) analysis, cerebellar NAA/Cr had the greatest AUC in differentiating MSA-C from SCA. The cutoff value of cerebellar NAA/Cr was 0.79 for raising an index of suspicion of MSA-C with a sensitivity of approximately 89.9%, a specificity of 88.0%, and an AUC of 0.939.

For patients with a similar disease duration in the bracket of 2–3 years, patients with MSA-C deteriorated faster with the need for walking assistance earlier in some of them^6^, and the prevalence of pontine HCBS was significantly higher in patients with MSA-C than in those with SCA. Significantly lower cerebellar NAA/Cr, Cho/Cr, and vermis NAA/Cr, Cho/Cr, and higher SARA scores were found in patients with MSA-C.

Using ROC curve analysis, cerebellar NAA/Cr had the best AUC differentiating MSA-C from SCA. The cutoff of cerebellar NAA/Cr was 0.75 for raising an index of suspicion of MSA-C with a sensitivity of approximately 86.9%, a specificity of 81.1%, and an AUC of 0.865.

For patients with a disease duration in the bracket of 4–5 years, patients with MSA-C had mostly been in a wheelchair^6^; the prevalence of HCBS was significantly higher in patients with MSA-C than in those with SCA. Significantly lower cerebellar NAA/Cr, Cho/Cr, and vermis NAA/Cr, Cho/Cr, and higher SARA scores were also found in patients with MSA-C.

Using ROC curve analysis, cerebellar NAA/Cr had the greatest AUC for differentiating MSA from SCA. The cutoff of cerebellar NAA/Cr was 0.72 for raising an index of suspicion of MSA with a sensitivity of approximately 90.3%, a specificity of approximately 84.2%, and an AUC of 0.917.

For patients with a disease duration in the bracket of 6–8 years, MSA-C patients had all been in a bedridden state^6^; the prevalence of HCBS was significantly higher in patients with MSA-C than in those with SCA. Significantly lower cerebellar NAA/Cr, Cho/Cr, and vermis NAA/Cr, Cho/Cr, and higher SARA scores were also found in patients with MSA-C.

Using ROC curve analysis, cerebellar NAA/Cr and Cho/Cr had similar AUCs in differentiating MSA from SCA. The cutoff of cerebellar NAA/Cr was 0.64 for raising an index of suspicion of MSA with a sensitivity of approximately 83.9%, a specificity of 87.6%, and an AUC of 0.905. The cutoff of cerebellar Cho/Cr was 0.54 for raising an index of suspicion of MSA with a sensitivity of approximately 80.7%, a specificity of approximately 92.8%, and an AUC of 0.913.

We only had 30 patients with a disease duration longer than 8 years in the MSA cohort. Although the disease durations were significantly longer in SCA patients, further subgroup analysis was not possible given the limited number of MSA-C cases with a disease duration longer than 8 years. The prevalence of HCBS was significantly higher in patients with MSA-C than in those with SCA. Significantly lower cerebellar NAA/Cr, Cho/Cr, and vermis NAA/Cr, Cho/Cr, and higher SARA scores were also found in patients with MSA-C.

Using ROC curve analysis, cerebellar NAA/Cr and Cho/Cr had similar AUCs in differentiating MSA from SCA. The cutoff of cerebellar NAA/Cr was 0.64 for raising an index of suspicion of MSA with a sensitivity of approximately 86.2%, a specificity of 87.0%, and an AUC of 0.908. The cutoff of cerebellar Cho/Cr was 0.56 for raising an index of suspicion of MSA with a sensitivity of approximately 87.5%, a specificity of approximately 83.3%, and an AUC of 0.904.

### Differentiation of MSA-C from SCA in patients without HCBS in the initial stages of disease (Table 4)

**Table 4:** The differences of parameters between HCBS negative patients with MSA-C and SCA with a disease duration within 1 year.

		Total (N = 61)	SCA (N = 46)	MSA-C (N = 15)	P value
Disease duration		0.90 ± 0.30	0.89 ± 0.31	0.93 ± 0.26	0.638
Cerebellum	NAA/Cr	0.89 ± 0.16	0.94 ± 0.13	0.74 ± 0.13	0.000
	Cho/Cr	0.72 ± 0.16	0.75 ± 0.16	0.64 ± 0.12	0.000
Vermis	NAA/Cr	0.84 ± 0.09	0.86 ± 0.08	0.77 ± 0.08	0.001
	Cho/Cr	0.67 ± 0.09	0.69 ± 0.09	0.62 ± 0.06	0.003

		Total (N = 51)	SCA (N = 39)	MSA-C (N = 12)	P value
SARA score		7.15 ± 4.49	6.40 ± 4.22	9.58 ± 4.64	0.039

Mann–Whitney test.

Numeric data are presented as mean ± standard deviation.

*HCBS* hot cross bun sign; *MSA-C* multiple system atrophy, cerebellar type; *SCA* spinocerebellar ataxia; *SARA* Scale for the Assessment and Rating of Ataxia; *NAA* *N*-acetyl aspartate; *Cr* creatinine; *Cho* Choline.

We found that pontine HCBS could mostly (89.16%) be detected during follow-up MRI studies in patients with MSA-C. However, discerning MSA-C from SCA in patients without pontine HCBS in the initial stages of disease is both challenging and important in terms of prognostication and genetic counseling. With a disease duration of < 1 year, we had 15 MSA-C and 46 SCA patients without HCBS in their MRI. Using ROC analysis, a significantly higher AUC of cerebellar NAA/Cr than cerebellar Cho/Cr was found for differentiating MSA-C from SCA. The cutoff for cerebellar NAA/Cr in these patients to raise an index of suspicion of MSA-C was 0.79. The sensitivity, specificity, positive predictive value (PPV) and negative predictive value (NPV) for MSA-C in patients without HCBS but having cerebellar NAA/Cr ≤ 0.79 were 73.3%, 90.0%, 71.0%, and 91.0%, respectively.

### Case demonstration (Fig. 1)

**Figure 1:**
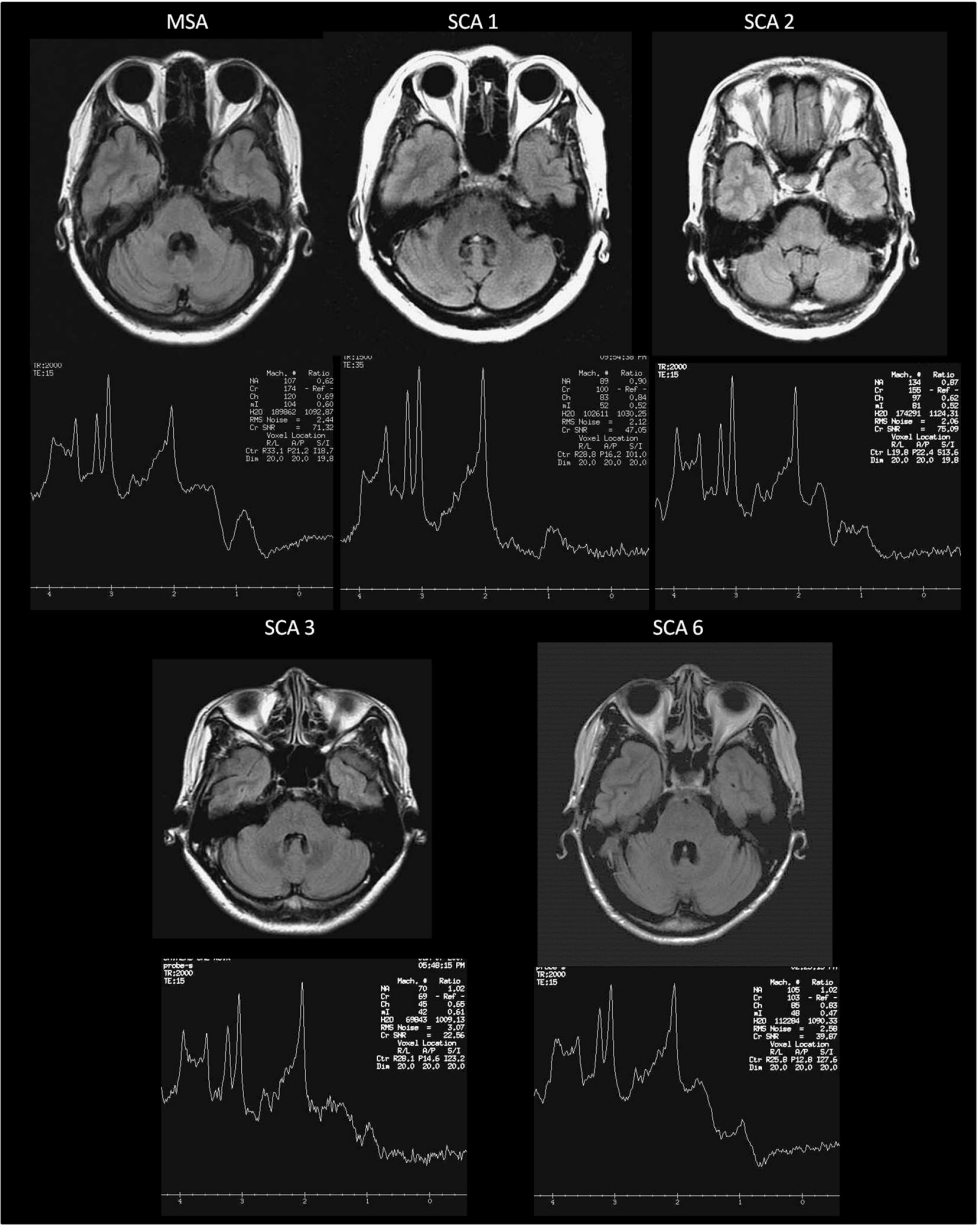
Axial FLAIR images at pons level and cerebellar MRS of patients with MSA or SCAs.

The cerebellar MRS of patients diagnosed as MSA-C and SCAs with a duration less than 1 year and without HCBS were demonstrated in Fig. 1. The SARA scores were 4, 9.5, 2, 4, 4 and cerebellar NAA/Cr were 0.69, 0.9, 0.87, 1.02, and 1.02 for MSA-C, SCA1, SCA2, SCA3, and SCA6, respectively.

## Discussion

In this study, the brain MRI and MRS features at different disease stages of MSA-C and SCAs were ascertained to gauge their discerning values. We found that patients with MSA-C had a higher prevalence of pontine HCBS, worse SARA scores, lower cerebellar NAA/Cr, cerebellar Cho/Cr, vermis NAA/Cr, and vermis Cho/Cr compared to those with SCA at corresponding disease stages, even in early encounters. For MSA-C patients with a disease duration < 1 year and without a pontine HCBS on the brain MRI, their cerebellar NAA/Cr, cerebellar Cho/Cr, vermis NAA/Cr and vermis Cho/Cr had already been lower than those with SCA. A cerebellar NAA/Cr ≤ 0.79 would be an optimal indicator of the possibility of MSA-C with a reliable accuracy (85.83%).

The HCBS could be used as a telltale sign for MSA-C with a high specificity and a high positive predictive value^7,8^. The grading of HCBS is related to disease severity^4^. Patients with a longer disease duration manifesting overt clinical symptoms would have more conspicuous pontine HCBS on brain MRI. However, other diseases causing gliosis of pontocerebellar fibers, such as dementia with Lewy bodies^9^, SCA^1^, or even progressive multifocal leukoencephalopathy^10^, could present with HCBS, as reported in the literature. Therefore, pontine HCBS alone may not be equally valuable at different disease stages. King reported that the PPV values of HCBS for MSA were 98.8% and 87.9% for patients with disease duration < 3 years and between 3–7 years, respectively^11^. By analyzing a larger sample size of patients, we further divided patients according to their disease durations, given that the lengths of disease durations could have a significant impact on the radiological features. The sensitivity of pontine HCBS to distinguish between MSA-C and SCA was found to be 82.35%, 93.14%, 96.97%, 96.83% and 96.67% at disease durations within 1 year, in the brackets of 2–3 years, 4–5 years, 6–8 years, and longer than 8 years, respectively. In this study, the pontine HCBS was only considered to be present when the intensity was above grade 3. The confidence for the presence of pontine HCBS was thus higher, but the sensitivity for early pontine HCBS could be lower. This might be the reason for the lower sensitivity of using HCBS to distinguish MSA-C from SCA in early disease stages.

Besides the pontine HCBS on brain MRI, we also found that the cerebellar and vermis NAA/Cr and Cho/Cr on the brain MRS were significantly lower in patients with MSA-C than in those with SCA with comparable disease durations. NAA is considered a marker for neuronal integrity, with a reduction of NAA/Cr in the brain representing neuronal loss or damage^12^. The Cho peak mainly denotes phosphocholine and glycerophosphocholine, compounds involved in membrane synthesis and degradation. Cr is used as the internal standard because its concentrations are relatively resistant to changes^13^. A reduction in Cho/Cr represents a decreased activity of membrane synthetic enzymes. Previous studies have reported lower NAA/Cr levels in patients with SCA and MSA^2,14–16^. The subtypes of SCA in our study, SCA1, 2, 3, 6, 7, and SCA17, are caused by the expansion of a CAG-repeat sequence in the respective genes, leading to abnormally long polyglutamine (Q) tracts in the encoded proteins^17^. The PolyQ-containing protein aggregation and misfolded protein deposition would subsequently lead to neuronal dysfunction and eventually cell demise, including loss of Purkinje cells and myelinated fibers with gliosis and synaptic loss in SCAs^18–20^. Oligodendroglial alpha-synucleinopathies are the characteristic neuropathological changes in MSA resulting in severe neuronal loss, gliosis, myelin pallor, and axonal degeneration^21^. The MRS features in the cerebellar hemispheres and vermis reflect the neuropathological changes in SCA and MSA and are evolving with time.

We found that cerebellar NAA/Cr could be a good and reliable radiological indicator of MSA in various disease stages. The cutoffs for cerebellar NAA/Cr were less than 0.79, 0.75, 0.72, 0.64, and 0.64 (with an AUC all greater than 0.86 with good sensitivities and specificities) at disease durations of within 1 year, and in the brackets of 2–3 years, 4–5 years, 6–8 years, and longer than 8 years, respectively. In patients who have a pontine HCBS on MRI but with higher cerebellar NAA/Cr ratios on the cerebellar MRS, SCA, rather than MSA-C, might need to be seriously considered (SCA2, in particular). Patients with MSA-C usually manifest a faster clinical deterioration, along with prominent dysarthria, autonomic dysfunction, and/or Parkinsonism, compared with those with SCAs. The significant destruction of the microenvironment of cerebellar hemispheres and vermis could be the reason and could, along with clinical symptoms, serve as an objective parameter for disease evaluation.

Of note is that at initial encounters and in the initial stages of disease, patients with MSA-C or SCA might have similar clinical presentation and MRI features, and some patients with MSA-C might not have conspicuous pontine HCBS on the MRI, but their cerebellar MRS is already significantly reduced. Microstructural changes in the cerebellum and vermis occur earlier and progress faster in MSA-C, even though the clinical symptoms remain mild.

In a recent study^22^, digenic inheritance of STUB1 variants and TATA-Binding protein (TBP) CAG triplet repeat expansions were reported in patients with SCA17 harboring 41–46 CAG repeat expansions. STUB1 variant are also shared by SCAR 16 and SCA 48. Sixteen patients (88.89%) in this study have a CAG repeat lengths between 43–46, two have a CAG repeat length of 51 in the TBP gene, and, regrettably, we have no data available at this moment about the STUB1 gene in these patients. However, two patients in our lab file were found to have STUB1 mutations and their CAG repeat lengths in the TBP gene were 37 and 38, respectively.

This study had some limitations. Although the study sample sizes are large compared with others in the literature, further subtype analysis of SCAs, beyond group analyses comparing groups of similar disease durations, were not possible given the constraints of small case numbers for each subtype. Second, we only compared SARA score as the sole clinical parameter in this study. A few other clinical parameters could have been used to evaluate ataxia if the study design was prospective. Future studies utilizing a prospective design with comprehensive clinical parameters in larger samples are warranted to validate the results.

## Conclusion

By using the pontine HCBS on brain MRI and cerebellar MRS, discerning MSA-C from SCA seems to be possible even in the initial stages of the disease. This study provides cutoff values of cerebellar NAA/Cr for use as the radiologic clues in differentiating MSA-C from SCAs.

## Data Availability

The data that support the findings of this study are available from the corresponding author upon reasonable request.
